# To the Rescue

**Published:** 1915-11

**Authors:** 


### To the Rescue.

The call of the eugenist has not been in vain. The ears of wealth have heard the far cry, realized its significance and is now putting into action the machinery that will lend a tremendous impetus to the work of race betterment. It is announced that Mrs. E. H. Harriman, widow of the deceased railroad magnate, will place her vast fortune to the task of popularizing the eugenic cause. No humanitarian endeavor has ever equalled this work of love. Not even the research work of Rockefeller can compare with this in the magnitude of its ultimate effects. For four years the way has been preparing for this work. This friend of the human race has been maintaining field workers all over the world who have accumulated valuable data. There has also been conducted a bureau of research at Cold Harbor Springs, L. I., the headquarters of the Eugenic Record Office.

The board that will have the supervision of this work consists of Alexander Graham Bell, inventor of the telephone, chairman; Dr. William H. Welch, pathologist at Johns Hopkins University; Dr. L. F. Barker, president of the National Commission of Mental Hygiene; Dr. L. H. Morgan, zoologist; Irving Fisher, professor of political economy at Yale, and Dr. E. E. Southard, famous pathologist of Boston. Dr. Charles B. Davenport is the director of the Eugenic Record Office and the Eugenic Society in Sing Sing. Investigation, so far, has shown that 10 per cent of the population of the United States are defective and should be prevented from procreating. The question of environment and heredity affecting the efficiency of the people, the cause of crime and its prevention are gone into with most encouraging results.

Not only negative eugenics, that is, the prevention of the unfit from multiplying, by imposing segregation and sterilization, but, positive eugenics, that is, race betterment by select breeding, or the encouragement of good mating, is a part of the program, and some definite practical suggestions are awaited with interest.

Some States have already passed sterilization laws which include New York, Michigan, Indiana, Wisconsin, Iowa, Kansas, Washington, California, Nevada, and North Dakota, while Pennsylvania, Nebraska, and Oregon have vetoed them. In one instance the Supreme Court ruled that such laws are unconstitutional, though the State of Washington holds that they are constitutional. While in some States the laws are not actively enforced, such a general and far-reaching work as this, instituted by this worthy woman, will go a long way in popularizing their adoption, not only in these States, that have enacted the laws, but will create the sentiment for similar legislation in other States.

